# Gibberellin Enhances the Anisotropy of Cell Expansion in the Growth Zone of the Maize Leaf

**DOI:** 10.3389/fpls.2020.01163

**Published:** 2020-08-04

**Authors:** Katrien Sprangers, Sofie Thys, Dagmar van Dusschoten, Gerrit T. S. Beemster

**Affiliations:** ^1^Research Group for Integrated Molecular Plant Physiology Research (IMPRES), Department of Biology, University of Antwerp, Antwerp, Belgium; ^2^Laboratory of Cell Biology and Histology, Antwerp Centre for Advanced Microscopy (ACAM), University of Antwerp, Belgium; ^3^IBG-2: Plant Sciences, Institute for Bio- and Geosciences, Forschungszentrum Jülich, Jülich, Germany

**Keywords:** anisotropic growth, cell expansion, gibberellin, kinematic analysis, leaf thickness, leaf width, maize, relative growth rate

## Abstract

Although plant organ shapes are defined by spatio-temporal variations of directional tissue expansion, this is a little characterized aspect of organ growth regulation. Although it is well known that the plant hormone gibberellin increases the leaf length/with ratio, its effects on cell expansion in the growing leaf are largely unknown. To understand how variations in rate and anisotropy of growth establish the typical monocotelydonous leaf shape, we studied the leaf growth zone of maize (*Zea mays*) with a kinematic analysis of cell expansion in the three directions of growth: proximo-distal, medio-lateral, and dorso-ventral. To determine the effect of gibberellin, we compared a gibberellin-deficient *dwarf3* mutant and the overproducing UBI::GA20OX-1 line with their wild types. We found that, as expected, longitudinal growth was dominant throughout the growth zone. The highest degree of anisotropy occurred in the division zone, where relative growth rates in width and thickness were almost zero. Growth anisotropy was smaller in the elongation zone, due to higher lateral and dorso-ventral growth rates. Growth in all directions stopped at the same position. Gibberellin increased the size of the growth zone and the degree of growth anisotropy by stimulating longitudinal growth rates. Inversely, the duration of expansion was negatively affected, so that mature cell length was unaffected, while width and height of cells were reduced. Our study provides a detailed insight in the dynamics of growth anisotropy in the maize leaf and demonstrates that gibberellin specifically stimulates longitudinal growth rates throughout the growth zone.

## Introduction

**Figure 1:**
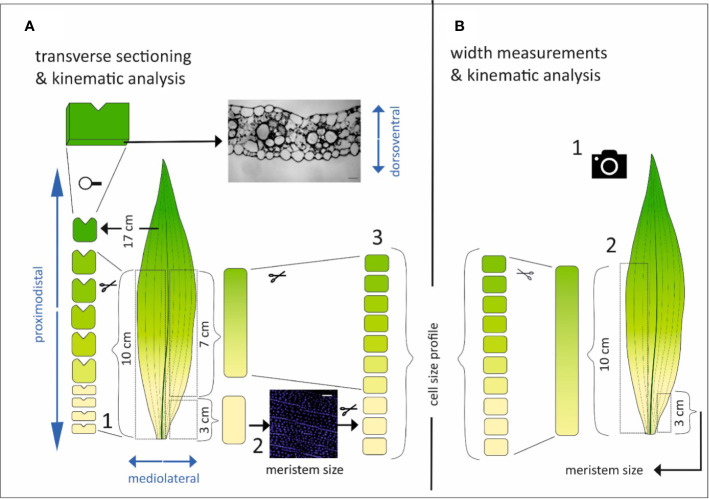
Schematic overview of the methodological approach. The different axes of the leaf are depicted in blue. **(A)** Three sets of leaves per genotype were harvested for transverse sectioning and kinematic analysis. The leaf was divided into two symmetrical parts by cutting along the midvein. One side of the leaf was cut at fixed positions from the leaf base (0.5, 1, 1.5, 2, 3, 4, 6, 10, and 17 cm) and the proximodistal orientation was marked by a small incision. At the opposite side of this incision cross sections were made (1). From the other part of the leaf, the basal most 3 cm were used for the quantification of the meristem size based on DAPI staining and fluorescence imaging of epidermal nuclei (2) and subsequently reused in combination with the following basal most 7 cm to cut in 1 cm pieces used for the determination of the cell size profile (3). **(B)** Three other sets of leaves per genotype were used to measure the leaf width by unfolding the leaf and take a picture with scale bar (1). Then, one side of the leaf was used to quantify the meristem size in the basal most 3 cm, while the other side was used to determine the cell size profile (2).

The characteristic elongated and flattened morphology of a monocotyledonous leaf suggests that, during its development, there must be large differences in growth along its three axes: proximo-distal (longitudinal), medio-lateral (width), and dorso-ventral (thickness), respectively (**Figure 1**). The orientation of these axes is established by oriented cell division patterns during leaf primordium formation (Hudson, 1999). The patterns of division and expansion along the longitudinal axis driving elongation growth after emergence from the whorl of older leaves have been extensively studied (Benhajsalah and Tardieu, 1995; Gázquez and Beemster, 2017). It is clear from the shape of the leaf and from previous investigations (Muller et al., 2007) that longitudinal growth is the dominant process in monocotyledonous leaf development. However, limited the control of spatio-temporal differences in the dynamics of cell division and expansion in the other directions are largely unknown.

In the dicotyledonous leaves of *Arabidopsis thaliana*, kinematic studies by Kalve et al. (2014) explained the establishment of the flattened round shape: During blade outgrowth expansion rates in the lateral direction are initially higher than longitudinal rates, but become equal at later stages of leaf blade development. Growth in thickness was an order of magnitude slower than longitudinal and lateral expansion. Low light reduced leaf thickness by specifically inhibiting dorso-ventral cell expansion rates. An earlier large-scale mutagenesis study in the same species revealed that lateral and longitudinal expansion are independently controlled (Berná et al., 1999). Studies on the root tip of *Arabidopsis thaliana* revealed that its elongate morphology is due to extremely anisotropic growth, where radial growth is absent, due to the radial orientation of cortical microtubules, thought to determine the deposition of cell wall microfibrils in the same orientation. Consistently, perturbing the orientation of the microtubules, using the microtubule inhibitor Oryzalin, partially released the restricted radial expansion rates and consequently, strongly increased root diameter (Baskin et al., 2004). In maize leaves, Muller et al. (2007) found a close correlation between the expression of specific expansin genes and longitudinal or lateral expansion rates.

Although these studies demonstrate the importance of growth anisotropy for (variations in) organ shape, it is still largely unclear how monocotyledonous leaves differentially regulate expansion in different directions in response to internal and external signals. These leaves essentially combine the linear spatial growth gradient similar to root tips with the lateral outgrowth of the blade seen in dicotyledonous leaves. The spatial distribution of growth defines the growth zone, which encompasses a division zone or meristem (where cells expand and divide, roughly maintaining a size equilibrium) and an elongation zone where cells only expand and, therefore, rapidly increase in size (Green, 1976). In monocotyledonous species, there have only been a few studies that addressed growth anisotropy. Maurice et al. (1997) described leaf shape and growth patterns of tall fescue (*Festuca arundinacea*) from emergence till maturity, showing that expansion in width and thickness occur mainly at the leaf base, while relative longitudinal expansion is dominant at a larger distance from the leaf base.

It is well known that gibberellin deficiency or insensitivity results in dwarf plants (Fujioka et al., 1988a; Fujioka et al., 1988b; Chandler and Robertson, 1999; Sakamoto et al., 2004; Otani et al., 2013). Overexpression of the rate limiting enzyme in gibberellin biosynthesis GA20 oxidase, on the other hand, results in taller plants (Nelissen et al., 2012). The effect of gibberellin on maize leaf length is attributed to altered dynamics of the cell division and expansion processes along the leaf blade. A local gibberellin peak establishes the transition between cell division and expansion, thereby determining cell production and ultimately leaf elongation (Nelissen et al., 2012). Wenzel et al. (2000) showed that gibberellin enhances longitudinal expansion and suppresses lateral and dorso-ventral expansion of epidermal pavement cells in the elongation zone of the barley leaf (*Hordeum vulgare*), leading to longer, narrower, and thinner leaves.

If gibberellin primarily stimulates cell division and expansion along the proximodistal axis, the more elongated leaf phenotype it induces can be explained by two distinct models. As a first possibility, expansion profiles along the growth zone are independently regulated for longitudinal, lateral, and thickness growth. If gibberellins specifically stimulate longitudinal growth, this would affect only the spatial distribution of longitudinal expansion, whereas expansion in lateral and dorso-ventral orientation would be unaffected. Alternatively, gibberellin could regulate the length of the growth zone for growth in all directions and increase anisotropy of growth, i.e., the ratio between rates of longitudinal vs lateral and dorso-ventral expansion rates. To address these distinct possibilities, we used a kinematic approach to quantify expansion rates in three directions in the growth zone of the maize (*Zea mays*) leaf. We compared wild type leaves with those of the *dwarf3* mutant that is deficient in gibberellin biosynthesis reducing the maximum concentration of the active GA1 in the growth zone from ca. 60 to 1 ng/g and the UBI::GA20OX-1 line that overproduces gibberellin, increasing these concentration to about 200 ng/g (Nelissen et al., 2012). In support of the second model, we found a simultaneous cessation of longitudinal, lateral, and dorso-ventral growth and stimulation of the size of the growth zone (for growth in all directions) by gibberellin. Gibberellin increased growth anisotropy by specifically stimulating longitudinal cell expansion in absence of an effect on expansion in lateral and dorso-ventral orientation.

## Materials and Methods

### Plant Material and Growth Conditions

We used segregating *dwarf3* seeds; d3-N660B (2008-414-2) in a W23xL317 wild type background; that are defective in the conversion of *ent*-kaurenoic acid to gibberellin12 early in gibberellin biosynthesis (Fujioka et al., 1988a) and the UBI::GA20OX-1 line, overexpressing the rate-limiting enzyme AtGA20-oxidase1 in gibberellin biosynthesis with its corresponding inbred B104 wild type (Nelissen et al., 2012). Plants were grown in pots in a controlled growth room (16-h day/8-h night, 25°C/18°C day/night, 300–400 µmol m^−2^ s^−1^ photosynthetically active radiation, provided by high-pressure sodium lamps). We analyzed the fourth leaf. First, after it emerged from the whorl of older leaves the length of this leaf (the distance from soil surface to the tip of the leaf) was measured with a ruler on three consecutive days. At three days after emergence, three leaves were randomly chosen for each genotype for cell length and leaf width measurements, and three other leaves were harvested for meristem size determination and transverse sectioning (**Figure 1**).

### Sample Preparation

Leaves for cross sections were spread on an agar plate that had a thin layer of water on top to prevent turgor loss. The leaf was divided by first cutting along the midvein. The first half was subsequently cut at fixed positions from the leaf base (0.5, 1, 1.5, 2, 3, 4, 6, 10 and 17 cm). A small incision marked the proximo-distal orientation of each section (**Figure 1A1**). The other half of the leaf was harvested for kinematic analysis according to Sprangers et al. (2016), whereby the three basal-most centimeters were harvested for DAPI staining and fluorescence imaging of epidermal nuclei to determine meristem length (**Figure 1A2**). The size of the division zone was determined as the distance from the leaf base to the most distal mitotic cells. After this, these sections were placed in lactic acid with the remaining parts of the leaf to measure the cell length and width profile. For this, the sections were whole-mounted using lactic acid as mounting medium (**Figure 1A3**). The UBI::GA20OX-1 leaves were too narrow to unroll without damaging the leaf. To reduce dissecting time and turgor loss, we therefore opted to dissect rolled leaves of this line for cross sectioning without cutting along the midvein first. This implicates that for these leaves there was no meristem size and cell size profile quantification. Therefore, the average cell length and width profile of UBI::GA20OX-1 plants harvested for leaf width measurements was used for kinematic calculations on this line.

For width measurements and kinematic analysis leaves were spread on an agar plate and a photograph (including a scale bar) was taken using a digital camera (Canon, D500; **Figure 1B1**). Subsequently, these leaves were used to measure the meristem size, cell length, and cell width profile as described before (**Figure 1B2**; Sprangers et al. (2016)). Since the UBI::GA20OX-1 leaves were difficult to unfold, they were first dehydrated with ethanol and then stored in lactic acid. This softens the leaves and made it easier to spread them to take a picture for leaf width measurements.

### Transverse Sectioning

Leaves were fixed overnight in Histofix (Carl Roth, Karlsruhe, Germany) at 4°C. After rinsing in 1X Phosphate Buffered Saline (PBS) solution (pH 7.4), leaves were gradually dehydrated in a series of ethanol (30%—2 h; 50%—2 h; 70%—2 h; 80%—2 h; 2× 90%—overnight; and 2 h, 3× 100%—1, 2, 3 h), gradually cleared in mixtures of 100% ethanol and xylene (3:1—overnight, 1:1—2 h and 3:1—2 h ethanol:xylene, 2× 100% xylene—2× 2 h) and subsequently impregnated with mixtures of 100% xylene and paraffin (3:1—overnight, 1:1—2 h, and 3:1—2 h xylene:paraffin). Finally, leaves were embedded in paraffin and 5-µm sections were made using a rotary microtome (RM2245; Leica, Nussloch, Germany). Sections were stained for 20 s in a 0.5% (w/v) aqueous toluidine blue staining solution, rinsed in water, and mounted under a cover slip using Entellan (Merck Millipore, Burlington, MA, USA).

### Morphometry

The whole leaf images of three plants per line were used to measure leaf width at 0.5 cm intervals starting from the leaf base, using Image J (Schindelin et al., 2012; Rueden et al., 2017). On whole mounted samples of five plants per line, epidermal cell length and width were measured at the same positions using image analysis software (Zen 2 Blue edition, Zeiss): The lengths of approximately 20 cells per position were measured in epidermal files immediately adjacent to the stomatal row. Epidermal cell width was measured for approximately 20 cells per position (including all cell types) that were dissected by a line perpendicular to the longitudinal axis of the leaf. On cross sections of three plants per line, total height of the cross section and height of approximately 40 adaxial and abaxial epidermal cells were measured and averaged. Damaged or collapsed cells were avoided. The thickness of the inner cell layers was determined as the difference between leaf thickness and the sum of the epidermal cell heights.

### Magnetic Resonance Imaging of the Maize Leaf Growth Zone

For imaging the leaf growth zone inside the sheaths of the older leaves, two W22 plants were selected three days after emergence of their 5^th^ leaf. The growth zone and surrounding older leaves were imaged at 5 cm from the soil surface, using a vertical bore 4.7T MRI system connected to a MR Solutions control system. To enhance sensitivity, a hand-wound solenoid (i.d. 1cm, 45° angle relative to B_z_, the magnetic field) was used as radio frequency coil. A spin echo sequence (Callaghan, 1991; TE = 8ms, TR=1.5s, FOV=10x10mm, image matrix = 256x256 with 10 slices, measurement time 1 h) was used.

To determine the thickness of the growing 5^th^ leaf first the noise level was determined and then the average signal intensity for those pixels in the middle of the leaves. Taking half of the sum of these two numbers yielded the signal cut-off. Pixels with an intensity below the cut-off counted as empty, those above as filled. This corrects for partial volume effects. A line plot perpendicular across at least 6 leaf segments was drawn with known length, the empty pixels were subtracted, and then divided by the number of crossed leaf segments. This yielded the average leaf thickness. The procedure was performed on 3 different images (slices) of each plant to yield a total of 9 measurements.

### Calculations

All organ and cell measurements were averaged and interpolated to 1-mm intervals using a local polynomial smoothing procedure (Rymen et al., 2010) to obtain the cell and organ size profiles. The length of the growth zone (*L*_gz_) was independently determined for each direction as the position distal from the base where the leaf/cell size equals 95% of the mature leaf/cell size in the smoothened organ/cell size profile. The mature cell size was calculated as the average of all cell sizes in the smoothened cell size profile distal to the growth zone.

The kinematic analysis in longitudinal direction was performed on the smoothened cell length profile according to Sprangers et al. (2016) to obtain the meristem length (*L*_mer_), cell cycle duration (*T*_c_), number of cells in the meristem (*N*_mer_) and elongation zone (*N*_el_). The leaf elongation rate (*LER*) was calculated as the change in leaf length between two successive daily observations divided by the time interval (ca. 24 h, depending on the exact time of measurement). The cell production rate (*P*) was calculated from the leaf elongation rate and mature cell length (*l_mat_*; mature cell length) according to Equation 1:

(Equation 1). $$P=\;\frac{LER}{l_{mat}}$$

Average cell division rate (*D*) was calculated as the ratio between cell production and the number of dividing cells (*N*_mer_; Equation 2).

(Equation 2). $$D=\;\frac{P}{N_{mer}}$$

Average cell cycle duration was then determined as the inverse of cell division rate multiplied by ln(2) to accommodate for the exponential nature of the division process:

(Equation 3). $$T_{c}=\;\frac{\text{ln}\left(2\right)}{D}$$

Residence time in the meristem (*T*_mer_) was calculated from the number of cells in the meristem and average cell cycle duration:

(Equation 4). $$T_{mer}=log2\left(N_{mer}\right)*T_{c}\;$$

In lateral and dorso-ventral dimensions we assumed that the length of the meristem and the time in the meristem equals that of the proximodistal axis. In the elongation zone the cell flux along the proximo distal axis (*F*) is constant and equals the cell production rate (*P*). Therefore, the time in the elongation zone (*T*_el_) was calculated as the ratio of the number of cells in the elongation zone (*Nel*) and flux (*F*):

(Equation 5). $$T_{el}=\frac{N_{el}}{F}$$

To calculate the number of cells expanding in width and thickness, the length of the growth zone based on leaf width and thickness was used. Within the meristem, the cell flux is not constant. Therefore, we used a linear regression [*F*=0 at the leaf base and *F*=*P* at the end of the meristem] to estimate the cell flux at any position the meristem. The cell flux (cells h^−1^) and the cell length (µm) were multiplied to calculate the velocity, i.e., the rate at which tissue moves away from the leaf base (*v*; µm h^−1^):

(Equation 6). v(x)=l(x)*F(x)

Assuming steady state growth, the relative leaf growth rate in length (RGR_Length_) within the meristem was calculated as the derivative of velocity with respect to distance from the leaf base (Baskin and Beemster, 1998):

(Equation 7). $$RGR_{Length}=\frac{dv}{dx}$$

with *v*(0) = 0 at the base of the leaf and at the end of the meristem (calculated as the product of *P* and cell length at the end of the meristem), respectively.

For calculations of relative leaf growth rate in length (RGR_Length_) and cellular relative growth rate in width (RGR_Width_) and thickness (RGR_Thickness_) we used the respective smoothened cell size profiles.

We also calculated the relative leaf growth rate calculations for width (RGR_Width_) and thickness (RGR_Thickness_) based on the smoothened organ size profile. For calculations of leaf level relative growth rates in width and thickness (RGR_Width_ and RGR_Thickness_) in the meristem we used the most basal position as ‘size 1' and the end of the meristem as ‘size 2' and *T_mer_* as *Δt* in:

(Equation 8). $$RGR=\frac{size2-size\;1}{\Delta t}$$

For the elongation zone RGRs we used the values at the end of the meristem and the end of the growth zone as ‘size 1 and 2', respectively, and *T_el_* as *Δt*.

## Results

### Maize Leaf Dimensions

**Figure 2:**
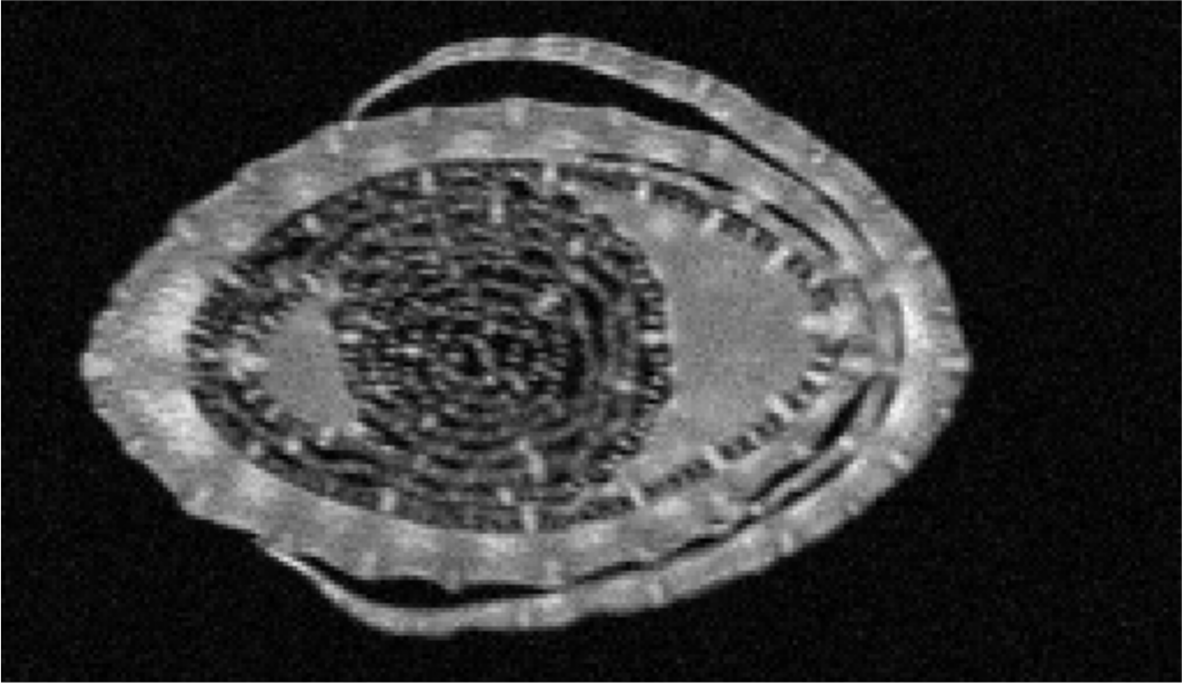
MRI scan through the pseudostem of a maize seedling. B104 seedlings were scanned at ca 5 cm from the base of the plant, three days after emergence of the 5^th^ leaf. The outer most leaf in the image is the second leaf, which is surrounding the third. Both are mature at this stage. The growing 4^th^ and 5^th^ leaves are curled up inside. Scale: Full image represents an area of 10x6mm.

**Figure 3:**
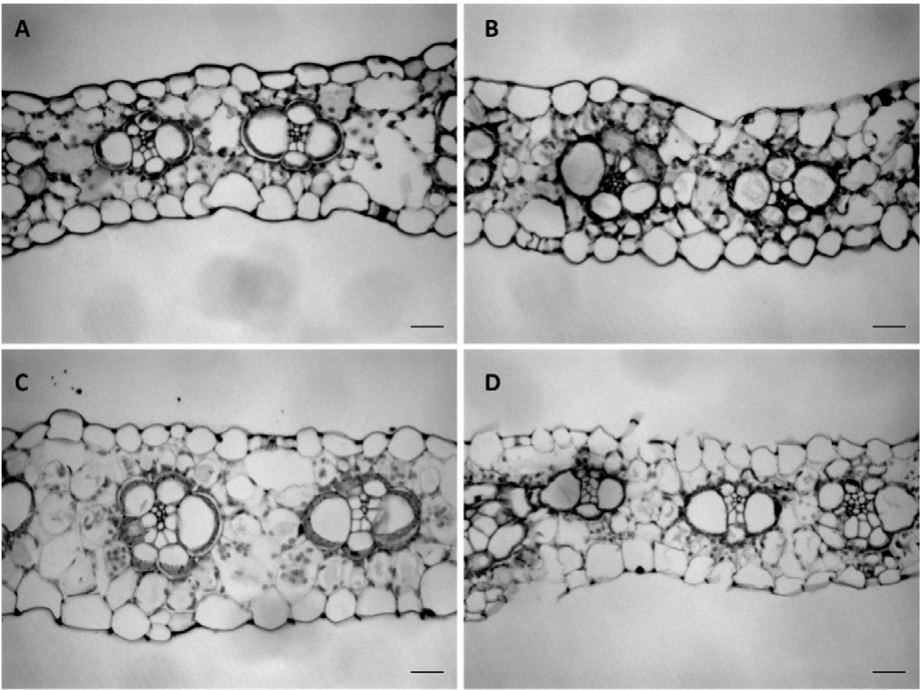
Transverse sections of the mature leaf blade of the fourth leaf at three days after emergence. **(A)** WT (W23xL317) of *dwarf3* **(B)** *dwarf3* **(C)** WT (B104) of UBI::GA20OX-1 **(D)** UBI::GA20OX-1. Scale bar: 20 µm.

To understand how cell growth is coordinated in the three main axes of growth (**Figure 1**), we quantified the overall dimensions of the growing 4^th^ leaf of maize seedlings, at 3 days after its emergence from the whorl of older leaves. At that time wild type leaves were 35 and 24 cm in length and 2.3 and 1.6 cm in width (W23xL317 and B104, respectively) so that their length is almost 15x greater than their width (**Table 1**). To determine blade thickness in situ, we performed Magnetic Resonance Imaging of the pseudostem of seedlings at 5 cm from the base of the leaves (**Figure 2**). The blades of the growing fourth and fifth leaves are much thinner than the sheaths of the surrounding second and third leaves, which are mature at this stage. Image analysis performed on nine different images from two different plants yielded an average thickness of the blade of 122 ± 10 μm. To obtain higher resolution images, we next analyzed cross sections on the emerged mature part of the fourth leaf (**Figure 3**). In good agreement with the measurements on the MRI images and values obtained by Muller et al. (2007), we measured a thickness of 113 ± 8 μm for B104, whereas the W23xL317 leaves were almost 20 μm thinner (**Table 1**). This means that the length of wild type leaves at this stage was 3 to 4 thousand times larger than their thickness, consistent with a strongly anisotropic growth.

**Table 1:** Leaf and cell dimensions of the 4^th^ leaf of maize plants 3 days after emergence.

Parameter	W23 x L317	*dwarf3*	%	p-value	B104	UBI::GA20OX-1	%	p value
Length								
Leaf (mm)	353 ± 10	141 ± 10	−60	<0.000	242 ± 25	364 ± 9	50	0.004
Epidermal cell (µm)	147 ± 3	152 ± 3	3	0.281	134 ± 3	138 ± 5	3	0.41
Width								
Leaf (mm)	23.3 ± 1.0	24.2 ± 0.9	4	0.631	15.7 ± 0.4	13.2 ± 0.7	−16	0.046
Epidermal cell (µm)	24.4 ± 1.2	29.4 ± 0.7	21	0.007	23.6 ± 0.9	19.7 ± 0.9	−16	0.026
Thickness								
Leaf (µm)	94 ± 11	111 ± 8	18	0.093	113 ± 8	96 ± 5	−15	0.033
Epidermal cell (µm)	18.1 ± 0.9	19.5 ± 0.5	8	0.210	18.5 ± 0.5	17.3 ± 0.6	−6	0.203
Leaf ratio								
Length/width	14.8 ± 1.2	5.6 ± 0.3	−62	0.001	13.3 ± 0.6	23.4 ± 0.8	76	<0.000
Length/thickness	4000 ± 100	1300 ± 100	−68	<0.000	2200 ± 200	3300 ± 200	51	0.012
Width/thickness	250 ± 30	220 ± 20	−12	0.45	140 ± 10	140 ± 10	0	0.99

The length and maximum width were determined from whole-leaf images. Thickness and epidermal cell size were measured on sections in the mature part of the growing leaf. Data are mean values ± SE (n = 5 for all data on length and epidermal cell width, n=3 for data all data on thickness and leaf width); p-values were calculated using a t-test. The percentage represents the difference of the mutant/transgenic line relative to the corresponding wild type.

### The Effect of Gibberellins on Leaf Shape

**Figure 4:**
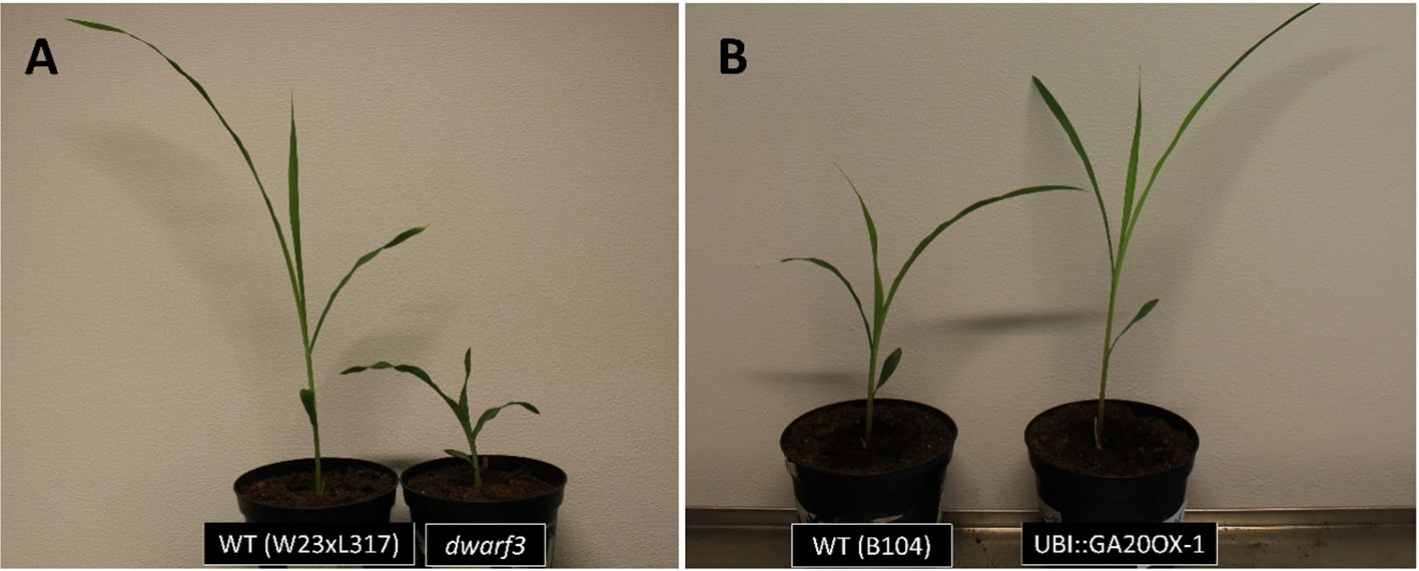
The phenotype of maize plants with altered gibberellin levels at three days after emergence of the fourth leaf. **(A)** The gibberellin-deficient *dwarf3* mutant and its wild type **(B)** The gibberellin overproducing UBI::GA20OX-1 line and its wild type.

We next investigated the effect of reduced and increased giberellin levels by comparing the dwarf3 mutant (Fujioka et al., 1988a) and the UBI::GA20OX-1 line (Nelissen et al., 2012) with their wild types (W23xL317 and B104, respectively). Consistent with earlier results (Chandler and Robertson, 1999; Sakamoto et al., 2004; Nelissen et al., 2012), gibberellins had a pronounced effect on plant morphology (**Figure 4**). Gibberellin deficiency in the *dwarf3* mutant reduced the length of the 4^th^ leaf by 60% and led to a small, but not significant, increase of its width and thickness (**Table 1**). Inversely, gibberellin overproduction in the UBI::GA20OX-1 line increased leaf length by 50% and had a small (ca 15%) negative effect on leaf width and thickness (**Table 1**). These results clearly show that gibberellin stimulated the overall anisotropy of leaf growth (**Table 1**).

### Spatial Distribution of Expansion in Length, Width, and Thickness

To investigate growth anisotropy, we evaluated the increase in each dimension along the developmental gradient with increasing distance from the base of the leaf. To this end we measured leaf width and epidermal cell length and width along the basal ten centimeters of the leaf on cleared leaves. We also measured the height of the epidermal cells and the inner tissues as well as overall leaf thickness on cross sections made at regular intervals along the leaf.

**Figure 5:**
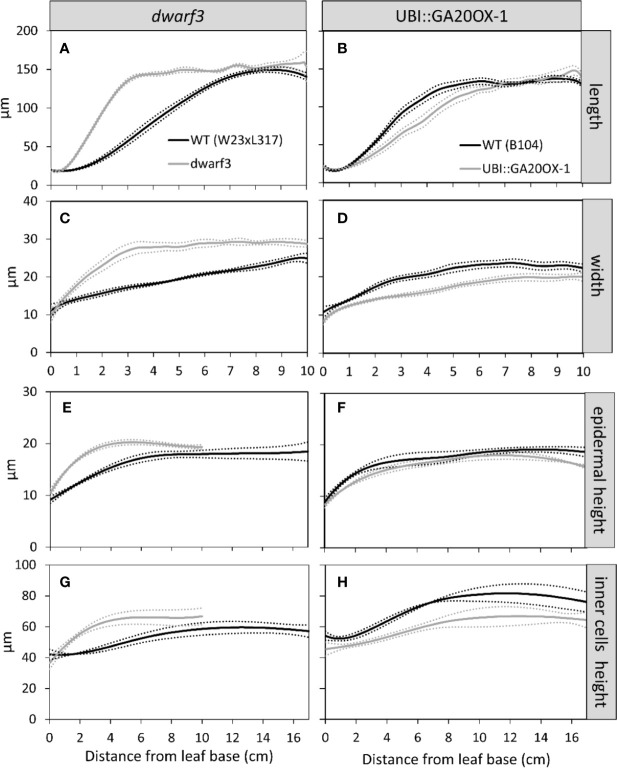
The effect of gibberellin on the cell size profile in length **(A, B)**, width **(C, D)**, and thickness **(E–H)**. Data are mean values ± SE with the mean given as solid line and the SE as dotted line (n = 5 for data on length and width, n = 3 for data on thickness).

**Figure 6:**
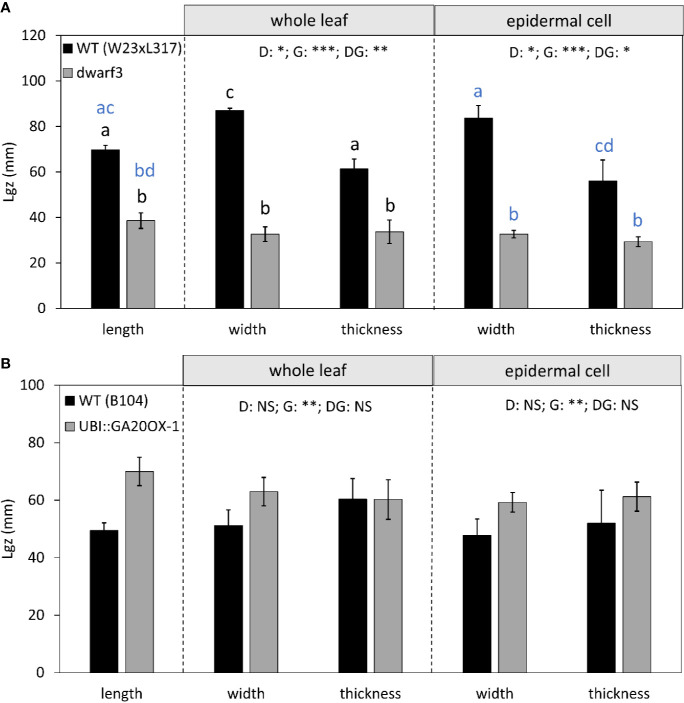
The effect of gibberellin on the length of the growth zone (in the longitudinal axis) for growth in length, width, and thickness. **(A)** *dwarf3* with its W23xL317 WT **(B)** UBI::GA20OX-1 line with its inbred B104 WT. Note that the longitudinal data at the whole leaf and epidermal level are both calculated based on cell length data and are, therefore, equal. They are presented in the left panel. The length of the growth zone in width and thickness were calculated based on whole leaf data (middle panel) and epidermal cell data (right panel). Data are mean values ± SE (n = 5 for data on length, n = 3 for data on width and thickness). A two-way ANOVA analysis was conducted as statistical test and significance (p value <0.05) for the two factors (direction [D] and genotype [G]) as well as their interaction [DG] is given on top of the panels. Only in [A] the interaction was significant, a Tukey's test was conducted to make pairwise comparisons that are depicted with letters above the bar charts, black for whole leaf data and blue for epidermal cell data. Significance codes: 0 ‘***' 0.001 ‘**' 0.01 ‘*' 0.05 ‘.' 0.1 ‘NS ‘ 1.

Cells typically reached their maximum length, width, and height within this 10-cm interval (**Figure 5**). However, in W23xL317 cell width did not reach a plateau (**Figure 5C**). These data allowed us to address the question if cell expansion in all dimensions is synchronized or stops at different distances from the leaf base. The length of the growth zone was derived from the cell size profiles (**Figure 5**) as the position where the cell size equals 95% of the mature value. For three of the four lines we found no significant differences in the size of the growth zone determined from the epidermal cell length width and thickness profiles (**Figure 5**). Independent estimations of length of the growth zone (*L_gz_*) based on whole leaf width and thickness profiles (**Figure 6**, middle panel) and epidermal cell size profiles (**Figure 6**, right panel) yielded very similar outcomes. In contrast to this general pattern, based on whole leaf measurements growth in width extended further than in length and thickness for W23xL317. Cell measurements showed the same pattern (**Figure 6A**). We conclude that, with exception of lateral growth in W23xL317, growth in all directions stops at the same position along the leaf base.

### Cell Length, Width, and Height Are Differently Affected by Gibberellin

We previously showed that for longitudinal cell expansion gibberellin increases the length of the growth zone (Nelissen et al., 2012), leading to the question if it has the same effect on growth in width and height. Therefore we investigated the cell size distributions of the *dwarf3* mutation and UBI::GA20OX-1 overexpression.

Consistent with earlier findings for cell length, *dwarf3* reached its mature cell length, width, and height at a shorter distance from the leaf base compared to its wild type. The UBI::GA20OX-1 line, on the other hand, had a longer growth zone compared to its wild type, although the differences are less pronounced than for *dwarf3* (**Figures 5** and **6**).

Although mature cell length does not differ from the wild type in both lines (**Figures 5A, B**), *dwarf3* has significantly wider mature cells compared to W23xL317, while the UBI::GA20OX-1 line forms more narrow cells than B104 (**Figures 5C, D**, **Table 1**).

Since it was not clear whether the epidermal cells in the UBI::GA20OX-1 line reached their maximum height at ten centimeter, we included a more mature part to determine the maximum cell size plateau (**Figures 5E–H**). *Dwarf3* showed a trend towards higher epidermal cells compared to its wild type (**Figure 5E**), whereas the UBI::GA20OX-1 line, had lower epidermal cell heights compared to its wild type, particularly in the most distal positions (**Figure 3F**, **Table 1**). In *dwarf3*, maximal thickness of the inner cell layers (mesophyll and bundle sheath cells) was marginally higher than that of the wild type (**Figure 5G**; **Figures 3A, B**). Interestingly, the thickness of the inner cell layers of the UBI::GA20OX-1 line was strongly reduced throughout the growth zone compared to the wild type (**Figure 5H**; **Figures 3C, D**).

In the gibberellin deficient mutant, the length of the growth zone was reduced to approximately half that of the wild type (**Figure 6A**). The opposite was true for the UBI::GA20OX-1 line, though the difference is less pronounced (**Figure 6B**). Importantly, both whole leaf (**Figure 6**, middle panel) and cell level analyses (**Figure 6**, right panel) show that in function of gibberellin levels the size of the growth zone covaried for growth in all three directions.

### Anisotropic Growth Is More Pronounced in the Meristem Compared to the Expansion Zone

Growth of the maize leaf is driven by two spatially separated processes: Proliferation in the division zone at the leaf base is responsible for cell production (cell growth in combination with division). Cell expansion (cell growth in absence of division) occurs in the elongation zone, immediately distal to the meristem and determines final cell size. Because our leaf and cell measurements spanned the entire growth zone, we could address the question if growth anisotropy is similar in meristematic and expanding cells.

**Figure 7:**
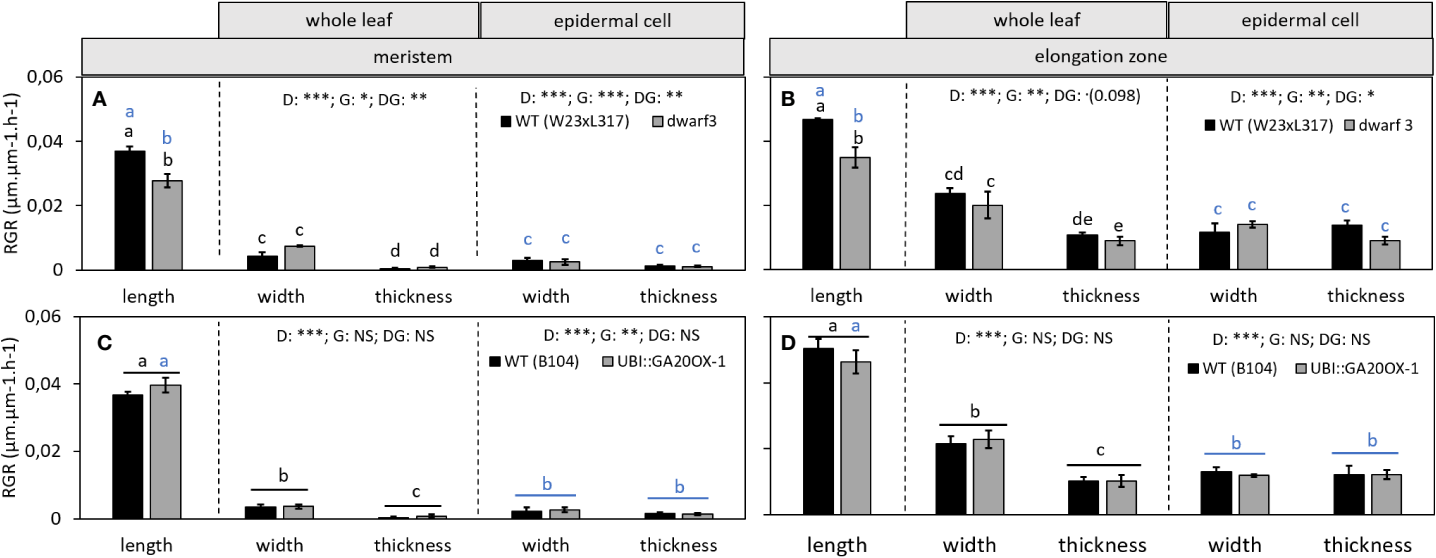
The effect of gibberellin on the relative growth rate (RGR) throughout the growth zone of the leaf. **(A, C)** The effect of gibberellin deficiency and overaccumulation of gibberellin, respectively, on the relative growth rate of the meristem in length, width, and thickness. **(B, D)** The effect of gibberellin deficiency and overaccumulation of gibberellin, respectively, on the relative growth rate of the elongation zone in length, width, and thickness. Note that in B and D, the longitudinal data at the whole leaf and epidermal level are both calculated based on cell length data and are therefore equal. They are presented in the left panel of B and D. Data are mean values ± SE (n = 5 for data on length, n = 3 for data on width and thickness). A two-way ANOVA analysis was conducted as statistical test and significance (p-value <0.05) for the two factors (direction [D] and genotype [G]) as well as their interaction [DG] is given on top of the panels. If the interaction was significant, a Tukey's test was conducted to make pairwise comparisons that are depicted with letters above the bar charts, black for whole leaf data and blue for epidermal cell data. If the interaction was not significant, growth in different directions were compared irrespective of genotype, as indicated by a horizontal bar. Significance codes: 0 ‘***'; 0.001 ‘**'; 0.01 ‘*'; 0.05 ‘.'; 0.1–1 ‘NS ‘.

Using fluorescence imaging of epidermal nuclei, we first determined that the meristem boundary was located at 9 and 14 mm from the base of the leaf in W23xL317 and B104, respectively (**Table S1**). This allowed us to calculate the average relative growth rates in the division and elongation zone for all the three directions (**Figure 7**). As expected, relative growth rates in proximodistal direction (RGR_length_) were consistently higher than growth rates in lateral and dorso-ventral orientation, in both the meristem and the elongation zone. Lateral expansion rates (RGR_width_) in turn were higher than dorso-ventral expansion rates (RGR_thickness_) (**Figure 7**). In the elongation zone, the growth rate in lateral direction (RGR_width_) was approximately half that of the longitudinal growth rate (RGR_lenght_) and dorso-ventral growth rates (RGR_thickness_) were only 25% of the longitudinal growth rate (**Figures 7B, D**, whole leaf). In the meristem, growth was more anisotropic: longitudinal growth (RGR_length_) was approximately seven-fold higher than that in width, while the dorsoventral growth rate (RGR_thickness_) was almost zero (**Figures 7A, C**, whole leaf). These results show that growth was more anisotropic in the meristem than in the elongation zone.

### The Degree of Anisotropy at the Leaf Level Is Not Reflected at the Cellular Level

To assess to what extent these leaf level measurements are reflected by the growth of individual cells, we separately calculated the relative cell growth rates (RGRs) for the epidermal cells from the cell size profiles. Overall cellular growth rates mirrored those determined at the whole leaf level (**Figure 7**). However, the difference between growth in width and thickness at the whole leaf level was not observed for epidermal cells. Epidermal cells grew equally fast in width as in thickness, while at the whole leaf level growth in width appeared to be faster than to growth in thickness both in the meristem and elongation zone (**Figure 7**).

### Gibberellin Changes the Anisotropy of Leaf and Cell Expansion

Next we assessed if the effect of gibberellin on growth anisotropy was specific for elongating or expanding cells. Localization of the meristem boundary by DNA fluorescence imaging confirmed earlier work (Nelissen et al., 2012), showing that *dwarf3* was inhibited in proximodistal growth because of a lower cell production rate due to a shorter meristem, containing fewer dividing cells. In our conditions, the increase of meristem length in the UBI::GA20OX-1 line was also observed (**Table S1**), but less pronounced as observed by Nelissen et al. (2012).

The effects of gibberellin on leaf length/width and length/thickness ratios were reflected in the growth rates in different directions: In the meristem and in the elongation zone of *dwarf3*, longitudinal expansion rates (RGR_length_) were significantly lower than in the corresponding wild type, while expansion in width (RGR_width_) and thickness (RGR_thickness_) was not significantly affected by the mutation (**Figures 7A, B**). This implies that growth of *dwarf3* was less anisotropic than its wild type. In the UBI::GA20OX-1 line longitudinal growth rates showed an opposite trend, albeit not significantly. Growth in width and thickness were also not significantly different from the wild type (**Figures 7C, D**). The absence of an effect of gibberellin on lateral and dorso-ventral growth was observed in both whole leaf and epidermal cell analyses (**Figure 7**, middle and right panels, respectively).

### Residence Time in the Elongation Zone Can Compensate for Differences in Growth Rate

**Figure 8:**
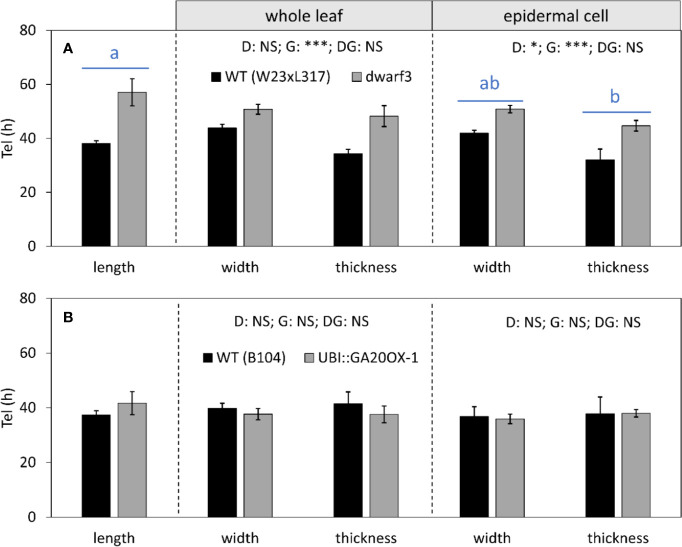
The effect of gibberellin on the time spent in the elongation zone (T_el_) in all directions. **(A)** *dwarf3* with its segregating W23xL317 WT **(B)** UBI::GA20OX-1 line with the inbred B104 WT. Values were calculated based on whole leaf data (middle panel) and epidermal cell data (right panel). Note that the longitudinal data at the whole leaf and epidermal level are both calculated based on cell length data and are, therefore, equal. They are presented in the left panel. Data are mean values ± SE (n = 5 for data on length, n = 3 for data on width and thickness). A two-way ANOVA analysis was conducted as statistical test and significance (p-value <0.05) for the two factors (direction [D] and genotype [G]) as well as their interaction [DG] is given on top of the panels. Because the interaction was not significant, a Tukey's test was conducted to make pairwise comparisons irrespective of genotype depicted with blue horizontal bars and letters above the bar charts for epidermal cell data. Significance codes: 0 ‘***'; 0.001 ‘**'; 0.01 ‘*'; 0.05 ‘.'; 0.1–1 ‘NS ‘.

Mature cell dimensions are not only determined by rate, but also by residence time in the elongation zone. In *dwarf3*, the reduced longitudinal growth rates (**Figure 7**) were compensated by a longer residence time in the elongation zone (**Figure 8A**), explaining the absence of an effect on mature cell length despite the reduced expansion rates (**Figure 5**). Because growth in all dimensions stops at the same position (**Figure 6**) and relative expansion rates in width and thickness are not affected by gibberellin (**Figure 7**), the increased time spent in the elongation zone also explains the enhanced height and width of cells at maturity (**Table 1**). In contrast, the duration of cell expansion in the UBI::GA20OX-1 line is similar to its wild type (**Figure 8B**), suggesting that more subtle changes cause the reduced height and width of its cells at maturity (**Figure 5**).

## Discussion

We quantified relative growth rates in three dimensions in both the division and elongation zone of the growing maize leaf and determined how gibberellin affects these rates. To make our studies feasible, we based our calculations on a steady-state assumption, essentially assuming width and thickness profiles to be stable over time. This allows to study a single time point, rather than 3 or more. Muller et al. (2007) show that for thickness this assumption is approximately true, whereas for growth in width there is a time-dependent contribution, that relatively limited, particularly in the meristem. Therefore, we can assume that our main conclusions would not be affected by adapting a more laborious non-steady state approach.

Our results demonstrate that growth in length is always dominant, but more anisotropic in the meristem compared to the elongation zone. This contrasts with previous results in tall fescue where relative growth rates in width and thickness were dominant at the leaf base, shifting towards longitudinal growth in the elongation zone (Maurice et al., 1997). This suggests that leaf growth patterns can differ between monocotyledonous species and/or growth conditions.

**Figure 9:**
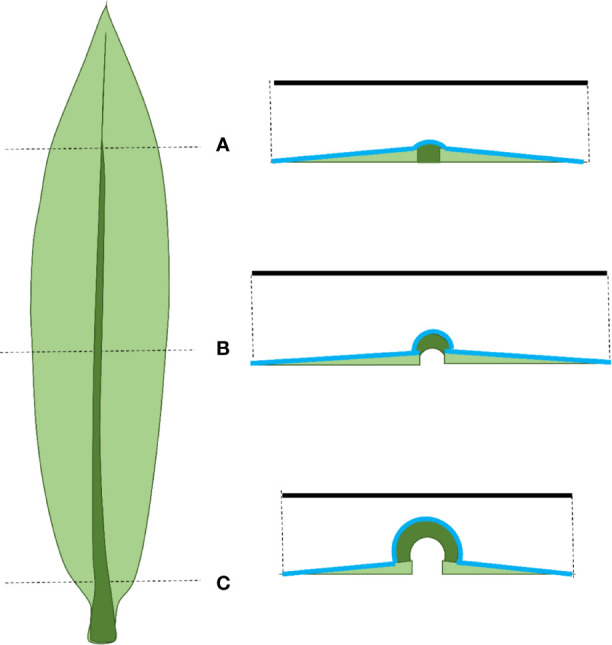
Schematic representation of a maize leaf on the left and cross-sections along this leaf on the right. The solid black line represents the measured width, the blue line represents the actual width. **(A)** At the tip of the leaf the mid vein is thin and the leaf is easily flattened making width measurements reliable. **(B)** At the end of the growth zone leaf flattening is relatively easy, making width measurements still reliable. **(C)** At the end of the meristem the mid vein is thick and rigid, making it hard to flatten the leaf. This leads to an underestimation of the width (difference between the length of the black and blue lines).

Our results demonstrate that anisotropic growth at the leaf level is not completely reflected at the cellular level. The difference in lateral and dorso-ventral relative growth rates observed at the leaf level was not found for the epidermal cells. This was primarily because relative growth rate in width at leaf level in the elongation zone was higher than that of epidermal cells (**Figure 7**). One explanation for this difference could be the formation of additional cell files by lateral divisions. Unfortunately, our analysis did not allow us to calculate division patterns in three directions. However, with the exception of divisions related to the differentiation of the stomatal complex, we observed very few lateral divisions when we analyzed the distribution of mitotic events. The transverse sections also suggested that all cell layers had been formed within the most basal five millimeter of the leaf (**Supplementary Figure 1**), while longitudinal division continues over a distance of approximately one centimeter (**Supplementary Table 1**). Therefore, it is possible that infrequent lateral division continue beyond the longitudinal division zone, contributing to leaf growth in width. Future studies mapping the division activity in different directions would provide an interesting addition to our observations of growth patterns in leaves. Another possible explanation for the difference in leaf- and cellular relative growth rates in width is the influence of the midvein at the base of the leaf on leaf width measurements. The relative leaf growth rate in width in the elongation zone is calculated based on leaf width at the beginning and end of this zone. The rigidity of the mid vein hinders leaf flattening, particularly at the base. Therefore, the measured width is probably underestimated at the beginning of the elongation zone (**Figure 9**), leading to an overestimation of the relative leaf growth rate in width at the leaf level.

Based on tall fescue leaf development, where growth in width and thickness occurred within and beyond the longitudinal growth zone (Maurice et al., 1997), we expected that the lateral and dorso-ventral growth zones in maize leaves would also extend beyond the longitudinal growth zone. While this was not observed, the W23xL317 wild type appeared to continue lateral growth beyond the longitudinal growth zone. However, our results for the 3 other genotypes showed growth zones of similar length for expansion in all directions, suggesting that this is either specific for this line or due to random biological variability.

The effect of gibberellin on anisotropic growth in monocot leaves has been studied before in the epidermal cells of the elongation zone of barley (Wenzel et al., 2000), where the relative growth rate in length and residence time in the elongation zone was not affected by gibberellin. In maize, however, we show that the relative growth rate in length is decreased in *dwarf3*, which is compensated by a longer time in the elongation zone. The resulting mature cell length is thereby not affected by gibberellin, confirming previous results on maize (Nelissen et al., 2012). Interestingly, in response to drought stress a similar compensation for reduced cell expansion rates by increased residence time in the elongation zone occurs (Avramova et al., 2015), suggesting the involvement of a size-control mechanism. In modelling root growth, cell size control could be explained by dilution of an initial amount of GA to below a critical threshold concentration (De Vos et al., 2014). This dilution could effectively explain the trade-off between rates and duration of growth to reach a given mature cell size.

However, in other monocotyledonous species gibberellin enhanced mature cell length in leaves, leaf sheaths or stem internodes (Matsukura et al., 1998; Wenzel et al., 2000; Otani et al., 2013). In the UBI::GA20OX-1 line, mature cell length in stem internodes was also increased (Nelissen et al., 2012). Thus, in all these cases gibberellin stimulated organ size, indicating there is a consistent effect at the whole organ level. However, the effect on cell division and on cell size control may vary between species and organs.

Although gibberellin did not appear to affect the relative cellular growth rate in width and thickness, the mature cells were significantly wider and higher in the mutant and tended to be narrower and less high in the UBI::GA20OX-1 line (**Figure 5**). This demonstrates that changes in growth anisotropy in itself do not fully explain final cell size, but that also the time spent growing at this rate plays a crucial role. Thus, to understand differences in final leaf or cell shape, the kinetic quantification of expansion should include both rates and duration.

Since turgor pressure is isotropic, anisotropic growth results from anisotropic mechanical properties of the cell wall. Our results show that growth anisotropy is largest in the meristem at the base of the leaf and decreases towards the mature region. This corresponds closely to the distribution of GA levels (Nelissen et al., 2012), suggesting GA positively regulates growth anisotropy. Our observation that reduced (*dwarf3*) and elevated GA levels (UBI::GA20OX-1), lead to a reduction and increase of growth anisotropy, experimentally demonstrate this functional relationship. This leads to the question what could be the downstream mechanism that mediates this effect of GA. The direction of anisotropy is often linked to the perpendicular orientation of the cellulose microfibrils to the main axis of growth (Green, 1980; Taiz, 1984; Cosgrove, 1997; Suslov and Verbelen, 2006). The alignment hypothesis states that the orientation of newly formed microfibrils is dictated by the orientation of cortical microtubules (Green, 1962; Bringmann et al., 2012). A lack of gibberellin can reorient cortical microtubules from a transverse towards a more randomized pattern in the elongation zone of barley leaves (Wenzel et al., 2000). Microtubule organization is regulated by gibberellin through a physical interaction between DELLA proteins and the prefoldin complex that mediates tubulin folding (Locascio et al., 2013). Moreover, microtubules can be stabilized by gibberellin (Mita and Shibaoka, 1984). Nevertheless, the onset of radial expansion (i.e., the combination of lateral and dorsoventral expansion) was observed in the basal elongation zone of gibberellin-deficient barley leaves, where the orientation of cortical microtubules was not affected. Cortical microtubules also became disorganized before the longitudinal relative elemental growth rate began to decline (Wenzel et al., 2000). Similar findings have been obtained for *Arabidopsis thaliana* hypocotyls, where the increased elongation resulting from a transient gibberellin application was not dependent on the transverse orientation of microtubules (Sauret-Gueto et al., 2012). This suggests additional mechanisms mediating the growth anisotropy induced by gibberellin may exist. One possible explanation is that in addition to the orientation of microfibrils, the length and tensile properties of these microfibrils influence directional cell expansion (Wasteneys, 2004). The length and by consequence also strength of the microfibrils would depend on microtubule organization. It is therefore possible that gibberellin acts more on the length of microfibrils than on their orientation. Our observation that gibberellin only affects longitudinal, but not lateral or dorso-ventral expansion rates in maize leaves points at mechanisms that affect wall loosening, particularly perpendicular to the orientation of microfibrils. It is known that gibberellin positively correlates with the expression and activity of xyloglucan endotransglycosylase (XET), an enzyme linked to loosening the bonds between adjacent microfibrils (Potter and Fry, 1993; Smith et al., 1996; Jan et al., 2004; Yue et al., 2016). Also the expression of β-expansin, a non-enzymatic wall protein involved in wall loosening by loosening the microfibril connections, was induced by gibberellin treatments in the internode of rice (Lee and Kende, 2001). Across different maize lines Muller et al. (2007) found a strong correlation between the expression of ZmEXPA4 and relative leaf widening rates, whereas the expression of ZmEXPA9 and ZmEXPB2 corresponded equally to elongation and widening rates. Thus, it is conceivable that gibberellin by differential regulation of expansins and other cell wall modifying enzymes stimulates separation rather than sliding of oriented microfibrils, which would explain the specificity of its effect on growth perpendicular to the main direction of microfibril orientation.

In conclusion, our study of the maize leaf growth zone demonstrates that growth in all directions stops simultaneously, i.e., the length of the proximodistal, lateral and dorso-ventral growth zone is equal and positively affected by gibberellin. Relative growth rates in length are always dominant, but the degree of anisotropy decreases in concert with GA levels from the division zone towards the elongation zone. Finally, gibberellin enhances growth anisotropy in both the division and elongation zone by specifically stimulating longitudinal growth rates.

## Data Availability Statement

The raw data supporting the conclusions of this article will be made available by the authors, without undue reservation.

## Author Contributions

KS generated all plant material, performed the light microscopy, image, and kinematic analyses, and wrote the manuscript. ST performed tissue fixation and embedding and assisted the sectioning. DD performed the MRI, and GB supervised the experiments and co-wrote the manuscript.

## Funding

This work was supported by a PhD fellowship from the Flemish Science Foundation (FWO, 11ZI916N) to KS; project grants from the FWO (G0D0514N); a concerted research activity (GOA) research grant, “A Systems Biology Approach of Leaf Morphogenesis” from the research council of the University of Antwerp; and the Interuniversity Attraction Poles (IUAP VII/29, MARS), “Maize and Arabidopsis Root and Shoot Growth” from the Belgian Federal Science Policy Office (BELSPO) to GB.

## Conflict of Interest

The authors declare that the research was conducted in the absence of any commercial or financial relationships that could be construed as a potential conflict of interest.
