# Supplementary material for: Gibberellin Enhances the Anisotropy of Cell Expansion in the Growth Zone of the Maize Leaf
Source: Front Plant Sci. 2020 Aug 4;11:1163. doi: 10.3389/fpls.2020.01163 (PMC7417610; doi:10.3389/fpls.2020.01163)
Supplement: Supplementary file 1 [file Image_1.pdf]

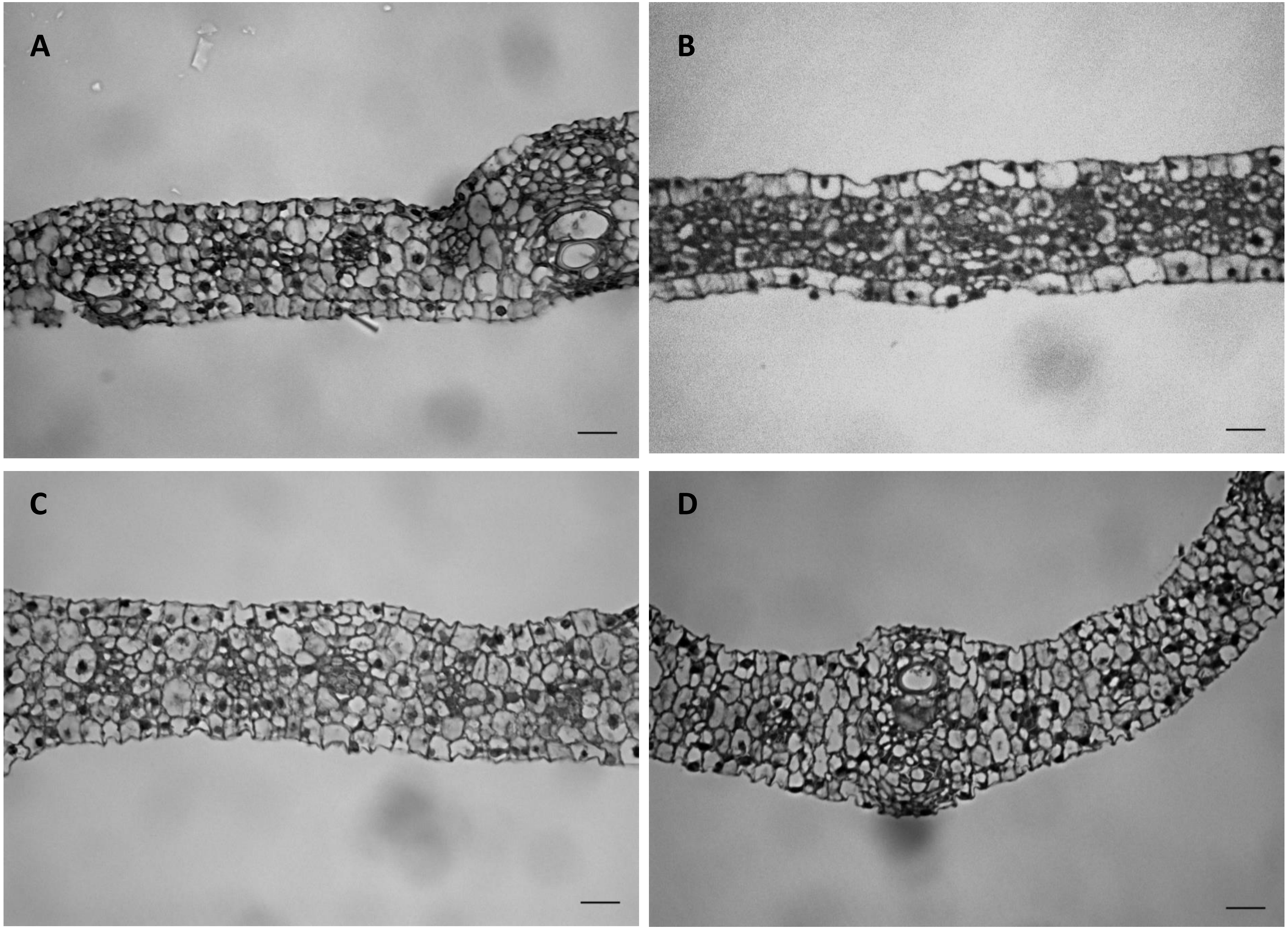

Figure S1. Transverse sections at five millimeter from the leaf base of the fourth leaf at three days after emergence. (A) WT of dwarf 3 (B) dwarf 3 (C) WT of UBI::GA20OX-1 (D) UBI::GA20OX-1. Scale bar: 20 µm.
