# Supplementary material for: Gibberellin Enhances the Anisotropy of Cell Expansion in the Growth Zone of the Maize Leaf
Source: Front Plant Sci. 2020 Aug 4;11:1163. doi: 10.3389/fpls.2020.01163 (PMC7417610; doi:10.3389/fpls.2020.01163)
Supplement: Supplementary file 2 [file Table_1.pdf]

Table S1. Kinematic analysis on cell division and cell expansion during the steady-state growth of the fourth leaf of dwarf 3 and UBI::GA20OX-1 with their corresponding wild types. Data are averages ± SE. Student's t-test was used as a statistical analysis and significance ($p < 0.05$) is depicted by stars. Parameters: Leaf Elongation Rate (LER), Mature cell length (L_{mat}), Cell production rate (P), Cell division rate (D), Cell cycle duration (T_c), Time in division zone (T_{div}), Length of the meristem (L_{mer}), Number of cells in the meristem (N_{mer}), relative cell elongation rate (R_{el}), Length of the cells leaving the meristem (L_{div}), Time in elongation zone (T_{el}), Number of cells in the elongation zone (N_{el}), Length of the growth zone (L_{gz}), Number of cells in the growth zone (N_{gz}). NS, not significant. The percentage represents the change according to the wild type.

Kinematic parameters	dwarf 3	WT of dwarf 3	%	t-test	UBI::GA20OX-1	WT of UBI::GA20OX-1	%	t-test
LER ($mm.h^{-1}$)	1.26 ± 0.06	3.30 ± 0.14	-61	***	3.23 ± 0.19	2.35 ± 0.09	38	***
L_{mat} (µm)	152 ± 3	147 ± 3	3	NS	138 ± 5	134 ± 3	3	NS
P ($cells.h^{-1}$)	8.3 ± 0.5	22.5 ± 1	-62	***	23.7 ± 2.1	17.7 ± 0.9	34	*
D ($cells.cell^{-1}.h^{-1}$)	0.030 ± 0.002	0.032 ± 0.001	-8	NS	0.042 ± 0.003	0.033 ± 0.001	25	*
T_c (h)	25 ± 2	22 ± 1	11	NS	17 ± 1	21 ± 1	-19	*
T_{div} (h)	207 ± 18	206 ± 7	-3	NS	155 ± 11	189 ± 7	-18	*
L_{mer} (mm)	5.5 ± 0.2	14.0 ± 0.4	-61	***	10.3 ± 0.4	9.0 ± 0.5	14	NS
N_{mer}	301 ± 28	707 ± 36	-57	***	568 ± 32	534 ± 34	7	NS
R_{el} ($µm.µm^{-1}.h^{-1}$)	0.040 ± 0.003	0.047 ± 0.001	-24	**	0.046 ± 0.004	0.050 ± 0.003	-8	NS
L_{div} (µm)	22.0 ± 1.2	24.8 ± 1.5	-9	NS	21.4 ± 1.8	20.7 ± 0.7	3	NS
T_{el} (h)	57 ± 5	38 ± 1	45	**	42 ± 4	37 ± 2	12	NS
N_{el}	465 ±14	861 ± 58	-46	***	970 ± 101	656 ± 31	48	*
L_{gz} (mm)	39 ± 3	70 ± 2	-44	***	70 ± 5	50 ± 3	41	**
N_{gz}	767 ± 25	1568 ± 94	-51	***	1538 ± 131	1190 ± 57	29	*
